# Real-time quantification of osteoclastic resorptive activity by electric cell-substrate impedance sensing

**DOI:** 10.3389/fcell.2022.921066

**Published:** 2022-08-19

**Authors:** Ineke D.C. Jansen, Thijs van Velzen, Teun J. de Vries, Robert Szulcek, Jack J. W. A. van Loon

**Affiliations:** ^1^ Department of Periodontology, Academic Centre for Dentistry Amsterdam (ACTA), University of Amsterdam and Vrije Universiteit Amsterdam, Amsterdam, Netherlands; ^2^ Department of Pulmonary Diseases, Amsterdam UMC, VU University Medical Center, Amsterdam Cardiovascular Sciences (ACS), Amsterdam, Netherlands; ^3^ Laboratory of in vitro Modeling Systems of Pulmonary and Thrombotic Diseases, Institute of Physiology, Charité—Universitätsmedizin Berlin, Corporate Member of Freie Universität Berlin and Humboldt-Universität zu Berlin, Berlin, Germany; ^4^ German Heart Center Berlin, Berlin, Germany; ^5^ Life Support and Physical Sciences Section (TEC-MMG), European Space Agency—European Space Research and Technology Centre (ESA-ESTEC), Noordwijk, Netherlands; ^6^ DESC (Dutch Experiment Support Center), Amsterdam University Medical Center Location VUmc, Amsterdam, Netherlands; ^7^ Department of Oral Cell Biology, Academic Centre for Dentistry Amsterdam (ACTA), University of Amsterdam and Vrije Universiteit Amsterdam, Amsterdam, Netherlands; ^8^ Department of Oral and Maxillofacial Surgery/Pathology, Amsterdam University Medical Center Amsterdam Bone Center (ABC), Amsterdam, Netherlands

**Keywords:** osteoclast, sensor, resorption, real-time quantification, bone, ECIS

## Abstract

In several diseases, bone resorption by osteoclasts is dysregulated. Thus far, no simple technique for real-time measurement of resorption is available. Here, we introduce an impedimetric bioassay for real-time monitoring of resorption by making use of the electrical insulating properties of the resorbable substrate calcium phosphate. Different chemical stimuli were applied to (pre)osteoclasts cultured on a layer of calcium phosphate in multi-well plates containing electrodes. By this, osteoclast activity can be measured continuously over days, and the effects of stimulating or inhibiting factors can be quantified. When cells were cultured in the presence of an inflammatory factor such as IL-1β, the resorptive activity started earlier. The measured decline in resistance was higher at culture day 5 than at cultures with M-CSF or M-CSF + RANKL (M-CSF norm. Resistance = 1, M-CSF + RANKL = 0.7, M-CSF + RANKL + IL-1β = 0.5). However, at day 11, this difference had nearly disappeared. Likewise, bisphosphonates were shown to inhibit osteoclastic activity. Our findings illustrate the importance of real-time monitoring; wherefore, this method has high potential not only for the study of osteoclast resorptive activity in the context of osteoclast function and diseases but also could find application in high-throughput drug-testing studies.

## 1 Introduction

Osteoclasts are multinucleated cells uniquely equipped to resorb bone (Gothlin and Ericsson, 1976). They arise from mononuclear hematopoietic cells from the monocyte lineage (Boyle et al., 2003). These osteoclast precursor cells fuse to form multinucleated osteoclasts in the presence of macrophage colony-stimulating factor (M-CSF) and receptor activator of NF-κB ligand (RANKL) (Jansen et al., 2012).

Bone resorption can occur in distinct modes. Some osteoclasts resorb bone in a stationary way, leaving behind round resorption pits, whereas other osteoclasts resorb bone while moving over the bone surface by which they form resorption trenches (Merrild et al., 2015; Søe and Delaissé, 2017).

For the visualization of bone resorption, various staining methods can be used for instance; toluidine, Coomassie Brilliant Blue (CBB), and hematoxylin (Notoya et al., 1993; Vesprey and Yang, 2016; Cao et al., 2017). These dyes stain proteins including the bone matrix proteins that are exposed due to osteoclast activity and left in the resorption pit. However, all these staining methods that are used to visualize and semi-quantitatively evaluate bone resorption require the removal of osteoclasts. In addition, with these methods, it is not possible to quantify the absolute amount of resorption since only the resorbed area and not the depth of the resorption pit can be measured. Some researchers use advanced microscopy techniques, such as scanning electron microscopy, confocal microscopy, or infinite focus microscopy, for the calculation of resorption pit depth and osteoclast morphology (Chambers et al., 1984; Goldberg et al, 2012; Boyde and Jones, 1991; Winkler et al., 2010; Pascaretti-Grizon et al., 2011), but for all these methods, the cultures must be terminated and the resorbed area can only be measured at the end of the *in vitro* culture period and not real-time during the entire experiment. On-line measurements of resorption hold great potential to advance our knowledge on osteoclast kinetics in physiology and pathology. It could provide answers in mechanistic studies on the timing of resorption, the mode of action of various agents that are known or that are suspected of interfering with osteoclast activity, or the development of novel substrate materials, scaffolds, or coatings for tissue engineering and orthopedic interventions. In an attempt to visualize osteoclast activity in real-time, Alsharif et al., have used luminescent nanocrystals which were incorporated in the calcium phosphate coating and taken up by the osteoclasts during resorption, which could be quantified by confocal microscopy (Alsharif et al., 2013). The advantage of this optical measurement is that individual cells can be traced. However, this technology requires two steps before a signal is generated. It requires matrix resorption followed by nanocrystal internalization by the osteoclast. Both processes could be rate-limiting steps in the final measurement. Also, the raw data need to be processed and mathematically modeled to quantify the final activity.

Alternatively, Lo, Keese, and Giaever (Lo et al., 1993) showed that Electric Cell-substrate Impedance Sensing (ECIS) is a versatile tool to quantify cell behavior electrically (Giaever and Keese, 1991; Lo et al., 1993; Lo et al., 1995; Szulcek et al., 2015). ECIS is an easy-to-use, non-invasive real-time measurement system to quantify several cell biological processes, such as barrier integrity, cell adhesions, motility, and migration and responses to drugs and toxins in a single, automated measurement with a high temporal resolution of seconds (Szulcek et al., 2014). The ECIS principle to measure impedance (complex resistance to current flow) makes it an interesting method for measuring osteoclast resorptive activity. In the present study, ECIS was used as a novel method to monitor and quantify osteoclast resorptive activity during culturing. Wells containing measurement electrodes were coated with calcium phosphate, causing a constant resistance to the flowing current. During culturing with activated osteoclasts, the impedance was monitored at several time points as a function of resorptive activity.

The results of this study allow for more efficient and accurate research into the relation between osteoclasts and the on- and off-switches of resorptive activity. With the ECIS method, the resorptive activity can be measured continuously, which allows to deduce the exact time when resorption starts. Importantly, ECIS is much easier to handle than the staining methods and less time-consuming. This holds an enormous translational value when using models for enhanced osteoclast activity in diseases like osteoporosis, Paget’s disease, or periodontitis, where the effect of medication on osteoclast activity can be monitored over time.

## 2 Materials and methods

### 2.1 Calcium phosphate coating

#### 2.1.1 Pre-coating

To prepare calcium phosphate coatings (CAP), the procedure of Leeuwenburgh et al. (2001) was followed. A pre-coating was formed in a 96-well plate containing electrodes (Applied Biophysics, Troy, NY, USA) using a 5x concentrated tyrode solution. The solution (500 ml) contains 20 g NaCl, 0.5g KCl, 0.67 g CaCl_2_*2H_2_O, and 0.53 g MgCl_2_*6H_2_O. To lower the pH to 2, 10 Ll of 1 M HCl was added, followed by 0.18 g Na_2_HPO_4_*2H_2_O and small portions of NaHCO_3_ (2.5 g in total). pH was maintained at 6.0 using 1 M HCl. The solution was sterilized using a 0.22-μm filter (Millipore, Amsterdam, Netherlands). 250 μL of this solution was added to every well, then the plate was sealed in a bag (stericlin, VPgroup, Feuchtwangen, DE), and placed in a 37°C incubation while gently shaking (150 rpm). The plate was incubated for 24 h and then washed three times with sterile demineralized water.

#### 2.1.2 Crystalline layer of calcium phosphate

A calcium phosphate supersaturated solution (CPS) was used to induce crystal growth on the pre-coated well plate. The solution was prepared by adding 20 ml 1 M HCl to 500 ml demineralized water after which 0.18 g Na_2_HPO_4_*2H_2_O, 4.0 g NaCl, 0.3 g CaCl_2_2H_2_O, and 3.0 g TRIS were added. pH was corrected to 7.4 using 1 M HCl. The solution was sterilized using a filter (Millipore). In the 96-well plate, 250 ul of this solution was added to every pre-coated well (step 2.1.1). The plate was sealed in a bag (stericlin, VPgroup) and kept at 37°C for 48 h. CPS was removed, and the plate was washed with sterile demineralized water and dried overnight in a laminar flow cabinet.

#### 2.1.3 Coated plate with HCl treatment

To investigate whether we can measure the gradual dissolvement of the CAP coating, HCl was added to the plate in an increasing concentration, and the resistance was measured in the ECIS machine for 130 min. HCl shifts the pH and thereby induces dissolution of the coating. The concentration series was 0.0025, 0.005, 0.0075, 0.01, 0.025, 0.05, 0.075, and 0.1N HCl. The series was added in triplicate, and the resistance was measured in the ECIS immediately after HCl addition.

### 2.2 Osteoclasts

#### 2.2.1 Isolation and culturing of osteoclast precursors from mouse bone marrow

For the isolation of osteoclast precursors, the bone marrow of mice (C57BL/6) was used. Animal experiments were approved by the animal welfare committee of the VU University (DEC nr. ACTA 2014–2). Tibiae were dissected and transferred in a petri dish containing 3 ml α-MEM (Gibco, Paisley, United Kingdom) + 10% FCS (HyClone, Logan, UT) + 1% PSF (Sigma, St. Louis, MO, USA) + 100 μl heparin (170 IE/mL; Leo pharmaceutical products BV, Weesp, Netherlands). The tibiae were crunched in a mortar containing 5 mL of α-MEM + 10% FCS +1% PSF. To isolate bone marrow, the cell suspension was aspirated through a 21G needle, filtered through a 70-μm pore-size cell strainer (Greiner Bio-One, Monroe, NC), and collected in a 50-ml tube. A MUSE count and viability kit (MCH600103, Merck, Darmstadt, Germany) was used to determine the concentration of cells. The cell concentration was adjusted to 1.3*10^6^ cell/mL, and 10^5^ cells were seeded per well. Osteoclasts were generated with 30 ng/mL M-CSF and 20 ng/mL RANKL (both from R&D Systems, Minneapolis, MI, USA) cultured for 11 days and refreshed twice, at days 3 and 7. To enhance the osteoclast resorptive activity, 10 ng/mL interleukin-1β (IL-1β; Sigma) or 10 ng/mL Tumor Necrosis Factor-α (TNF-α; R&D systems, Minneapolis, MI) was added directly to the culture medium in combination with M-CSF and RANKL, as mentioned above (Cao et al., 2016; Cao et al., 2017; Wang and He, 2020). To inhibit osteoclast activity, 20 ng/mL pamidronate (Sigma) was added to the cultures.

#### 2.2.2 Isolation and culturing of osteoclast precursors from human blood

CD14^+^ blood monocytes as osteoclast precursors were isolated from human peripheral blood mononuclear cells (PBMCs) (ten Harkel et al., 2015). Briefly, blood from a buffy coat (Sanquin, Amsterdam, Netherlands) was diluted with PBS containing 1% citrate (1:2) and spun down (800 g for 30 min, without brake) in lymphoprep (Elitech, Puteaux, France) gradient solution. The resulting interphase containing peripheral blood mononuclear cells (PBMCs) was collected and washed with 1% citrate in PBS before it was passed through a cell strainer (40 µm Greiner Bio-One Monroe, NC) to ensure the recovery of a pure mononuclear cell population. The cells were counted (Muse cell counter, Merck, Darmstadt, Germany), and the cell pellet was resuspended in 80 µL buffer (PBS containing 0.5% BSA and 2 mM EDTA) for 10^7^ cells. Twenty µL of CD14-magnetic beads was added to this cell suspension (MACS microbeads, Miltenyi Biotech, Bergisch Gladbach, Germany). According to the manufacturer’s instructions, the cells and CD14-beads were mixed and incubated for 15 min at 4°C. The LS column was placed in the magnetic field, rinsed, and subsequently, the cell suspension was applied to the column. Unlabeled cells will pass through. Then, the column is removed from the magnet and CD14^+^ cells were flushed out and collected.

The isolated CD14^+^ cells were plated on a calcium phosphate–coated ECIS 96-well plate at a density of 10^5^ cells per well. Cells were cultured for 21 days in α-MEM (Gibco, Paisley, United Kingdom) supplemented with 10% FCS (HyClone, Logan, UT), 100 U/mL penicillin, 100 μg/ml streptomycin, and 250 ng/mL amphotericin B (Antibiotic Antimycotic solution, Sigma, St. Louis, MO) and for an additional 3 days with 25 ng/ml human recombinant M-CSF (R&D Systems, Minneapolis, MN) to induce differentiation. After these 3 days, the concentration of M-CSF was reduced to 10 ng/mL and combined with 2 ng/mL recombinant RANKL (R&D systems) till the end of the culture period. During culture, the cells were maintained at 37°C and 5% CO_2,_ and the culture medium was refreshed every 3–4 days for a period of 21 days. To enhance the osteoclast resorptive activity, 10 ng/mL interleukin-6 (IL-6; R&D) was added directly to the culture medium.

### 2.3 ECIS

The osteoclast precursors were cultured in a CAP coated 96-well plate containing electrodes (96W10idf PET, Applied Biophysics, Troy, USA), maintained at 37°C and 5% CO_2,_ and put in the ECIS (ECIS Z0 and 96W Array station, Applied Biophysics, NY, USA) where the resistance was measured at 64 kHz every 15 min. Data were registered using ECIS software. For detailed description and introductory videos, refer to (Szulcek et al., 2014). Representative data are shown at 64 kHz.

### 2.4 Osteoclast visualization

#### 2.4.1 Tartrate resistance acidic phosphatase staining

When the mature osteoclasts were formed after 11 days for mouse cells and 21 days for human cells, the osteoclasts were visualized with TRAcP staining. The commercially available leukocyte acid phosphatase kit (Sigma-Aldrich, St Louis, MD, USA) was used following the manufacturer’s instructions. In short, the wells were washed once with PBS and subsequently fixated with 4% formaldehyde in 0.1 M phosphate buffer (pH = 7.2) for 10 minutes at room temperature and subsequently washed once again with PBS and stained for TRAcP. The incubation time was 15 min for mouse cells and 60 min for human cells in the dark at room temperature. Subsequently, the nuclei were stained with 4′,6-diamidino-2-phenylindole (DAPI) and examined with an inverted fluorescence light microscope equipped with a digital camera (DM IL, Leica microsystems, GmbH, Wetzlar, Germany) using phase contrast for TRAcP staining and fluorescent light to visualize the nuclei.

#### 2.4.2 Scanning electron microscopy

The area around the electrode lysed by osteoclasts was visualized with a scanning electron microscope (LS15 Zeiss, Oberkochen, Germany) at a magnification of ×2000. The bottom of the 96-well plates with electrodes was cut out with biopsy stans (Microtek, Mosta, Malta), dried in a vacuum oven (Vacutherm, Heraeus, Thermo Scientific, Waltham, MO, USA), and gold-sputtered for electron conductivity.

### 2.5 Statistical analysis

Data analysis was performed using GraphPad Prism 8 (GraphPad, San Diego, CA). One-way ANOVA was used to analyze differences between three or more groups at different time points. A paired t-test was used to analyze differences between two groups at different time points. The number of replicas was n = 6, unless indicated differently. Groups were considered significantly different when *p* ≤ 0.05.

## 3 Results

### 3.1 Increasing concentrations of HCl result in gradual dissolving of the CAP coating

**FIGURE 1:**
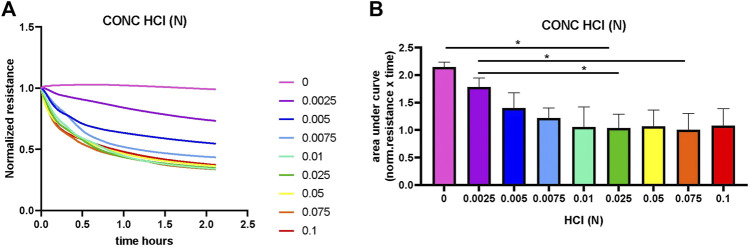
Dissolving of CAP by titration of HCl concentration. HCl was added in increasing concentrations to dissolve the calcium phosphate coating (CAP) on electrodes containing 96-well plates. Resistance was measured by ECIS at a frequency of 64 kHz for 2 hours. **(A)**. Overview of the normalized resistance for all HCL concentrations. In control with no HCl, CAP coating is not lysed and resistance not changed. The higher HCL concentrations had dissolved the CAP coating already at very early time points. **(B)**. Quantification of area under the curve shows significant differences (*p < 0.01 n = 3).

To assess whether an increase in conductivity was indeed the result of lysis of the CAP coating, an experiment was conducted with HCl-induced lysis of CAP (Figure 1). The dissolving of the CAP coating with gradually increasing concentrations of HCl started directly after addition to the culture well. After adding the HCl, the resistance was measured immediately in the ECIS. However, while placing the plates in the ECIS, some of the coatings – especially with the higher HCl concentration – already started to dissolve. This is the reason why the resistance was not the same for all the wells at t = 0. As such, 0.1, 0.075, 0.05, 0.025, and 0.01N HCl (Figure 1A) had already dissolved the CAP coating at the start of measurement, resulting in a lower normalized resistance. This gradually became less with a lower concentration where we could follow dissolution over time. The 0 and 0.0025N HCl started with high resistance, meaning that the CAP is not dissolved and decreases more slowly over time. Calculation of the area under the curve (Figure 1B) showed that significant differences exist between the two lowest concentrations (and 0.0025N HCl) and 0.025 and 0.075N HCl.

### 3.2 Osteoclasts resorb calcium phosphate on ECIS plates

**FIGURE 2:**
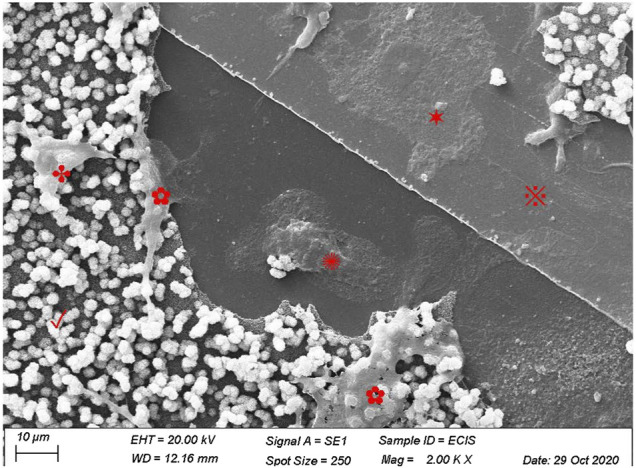
Human osteoclasts on CAP-coated ECIS plates. Human CD14+-cells were cultured for 21 days with M-CSF and RANKL and visualized by scanning electron microscopy. The image shows a partly intact CAP (✓) layer on the culture surface of ECIS wells. Some osteoclasts have lysed the CAP coating, exposing the electrode (※). Several osteoclasts were present on the bare multi-well plastic substrate (✺), on the electrode (✶), as well as osteoclasts that partially lysed CAP (✿), and an osteoclast still on top of the CAP coating (✤).

Before correlating the presence of CAP-lysing osteoclasts and increased conductivity, it must be established that osteoclasts indeed lyse the CAP that was formed on the ECIS plates. Using SEM, we were able to show that osteoclasts are present on the CAP-coated ECIS plate. Osteoclasts were present in the CAP removed area, indicating that these osteoclasts were responsible for resorption. Cells were also visible on top of the CAP layer (Figure 2).

### 3.3 Stimulatory molecules contribute to more resorption of CAP

**FIGURE 3:**
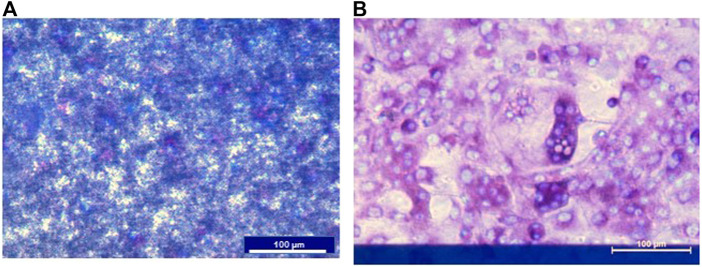
ECIS plate with CAP coating and mouse bone marrow cells cultured with M-CSF or M-CSF and RANKL. **(A)** Cells were cultured on an ECIS plate with CAP for 11 days with only M-CSF. No osteoclasts were formed, and the CAP coating is not lysed. Nuclei of the mononucleated cells were stained with DAPI (blue). **(B)**. When cultured for 11 days with M-CSF and RANKL multinucleated and TRAcP (purple), positive osteoclasts were formed. Nuclei stained with DAPI (blue). The blue horizontal bar in B is the ECIS plate electrode.

**FIGURE 4:**
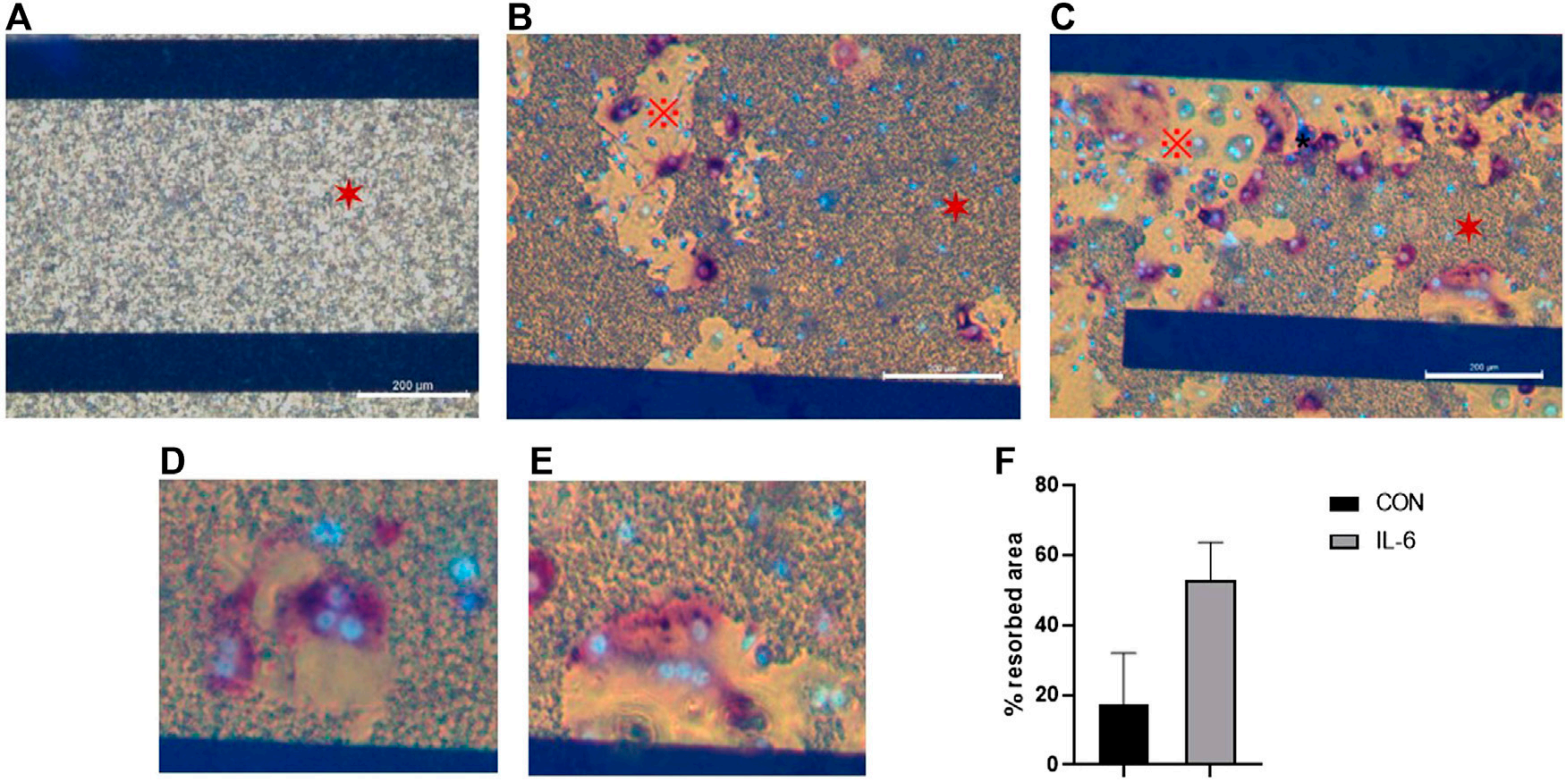
ECIS plate with CAP coating visualized with an inverted microscope. **(A)**. When no cells were present, the CAP coating (✶) is completely unaffected. **(B)**. Cells were cultured for 21 days with M-CSF and RANKL, and osteoclasts from human CD14+-cells were formed and able to dissolve the CAP coating. Osteoclasts were stained for TRAcP (purple) and nuclei with DAPI (blue). **(C)**. Osteoclasts were activated with IL-6 (i.e., M-CSF + RANKL + IL-6), resulting in higher dissolving activity. Osteoclasts were stained for TRAcP (purple) and nuclei with DAPI (blue). CAP coating (✶), dissolved area (※). **(D,E)** Higher magnification to illustrate that osteoclasts are located at the periphery of the CAP coating, where resorption takes place. **(F)** Percentage of the resorbed area in cultures with M-CSF + RANKL (=CON) and M-CSF + RANKL + IL-6 at day 21. Blue horizontal bars are the ECIS plate electrodes. Scale bar A-C = 200 µm.

Preosteoclasts adhered normally to the CAP-coated ECIS plates (Figure 3). Without RANKL, no osteoclasts were formed. Only mononuclear cells were present, and the CAP coating was not lysed (Figure 3A). In cultures with M-CSF and RANKL, osteoclasts were formed, and the CAP coating was partly resorbed (Figure 3B). In Figure 4A a micrograph of the coated ECIS plate without cells and the CAP coating was not lysed. When the cells were cultured with M-CSF and RANKL, TRAcP-positive osteoclasts were formed and the coating was partly lysed, osteoclasts being present at the periphery of the lysed areas (Figure 4B,D). When one of the inflammatory cytokines such as IL-6 was added to the cultures in combination with M-CSF and RANKL, more TRAcP-positive osteoclasts were formed and more of the CAP area was resorbed (Figure 4C,E). In Figure 4F, the resorbed area of control and IL-6 is quantified. However, the percentage of the resorbed area in the triplicate wells is not significantly different (n = 3).

### 3.4 Stimulatory or non-stimulatory molecules contribute to a specific time-dependent resorption

**FIGURE 5:**
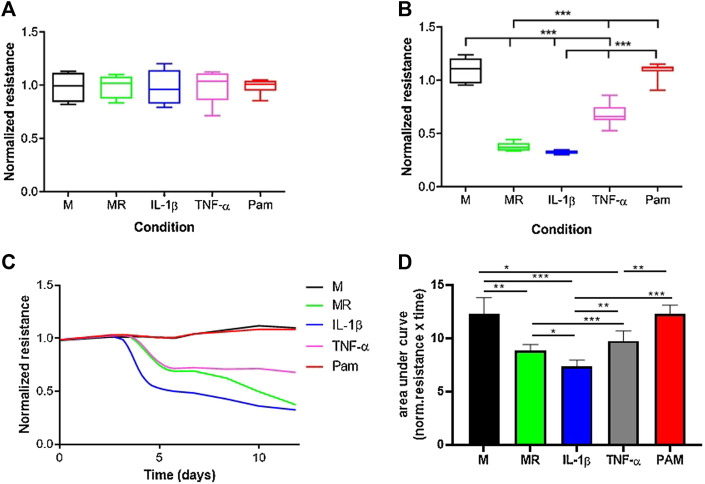
Real-time resorption quantification provides mechanistic insights into osteoclast formation and resorptive activity. M = medium with M-CSF, MR = medium with M-CSF and RANKL, IL = medium with M-CSF, RANKL and IL-1β, TNF-α = medium with M-CSF and RANKL and TNF-α, PAM = medium with M-CSF and RANKL and Pamidronate. Osteoclasts were generated from mouse bone marrow, and the resistance was measured for 11 days. **(A)**. No differences in resistance were found between the various conditions at timepoint zero since pre-osteoclasts were not differentiated into mature cells yet. **(B)**. After 11 days of culture, osteoclasts were formed and CAP coating was lysed, resulting in differences in resistance. Osteoclasts were formed in cultures with MR, IL-1β, and TNF-α, resulting in a lower resistance due to lysis of the CAP coating. In the cultures with only M or Pam, no active osteoclasts were present and no decline in resistance was recorded. (one -way ANOVA n = 6, ***p < 0.05). **(C)**. Overview of the resistance from day 0 to day 11. Typical for the lysis of CAP coating is the lag phase between days 0–4, where the resistance is not changed. Thus, no lysis has taken place because osteoclasts were not formed yet. From day 4, unique CAP lysis patterns emerge, likely due to differential activation of osteoclasts by MR supplemented with or without IL-1β or TNF-α, all leading to lower resistance. In the M and PAM cultures, no lysis occurred, likely due to the absence of osteoclasts, resulting in no change in resistance. **(D)**. Quantification of area under the curve shows significant differences (*p < 0.01, **p < 0.001, and ***p < 0.0001 n = 6) for most of the incubations. Only the incubations in which no osteoclasts were formed (M and PAM) show an equally high area under the curve. The incubation with IL-1β has the smallest area, which means that CAP lysis was the highest.

The normalized resistance was measured at timepoint = 0 for cultures with only M-CSF, M-CSF and RANKL, M-CSF, and RANKL with either IL-1β, TNF-α, or Pamidronate (Figure 5). At t = 0, the normalized resistance is comparable for all cultures because no osteoclasts are formed yet and the coating is still intact (Figure 5A). As expected, when only M-CSF was added to the cultures the resistance stayed high in these cultures since no osteoclasts were formed, and the CAP coating was not lysed (Figure 5B). In the presence of M-CSF and RANKL with or without IL-1β, the resistance decreased to a comparatively low value on day 11, representing the endpoint measurement. Interestingly, real-time recording (Figure 5C) revealed that IL-1β induces osteoclast activity earlier in time (day 4 *vs*. day 5) and accelerates the lysis of calcium phosphate when compared to MR alone. Another unique pattern was observed when adding TNF- α, where a plateau of resistance was reached at a different level when compared to MR or MR + IL-1β (0.8 *vs*. 0.4; *p* < 0.05 n = 6). These real-time recordings show thus far unknown calcium phosphate lysis patterns that could be unique to the stimulus that was given and could provide novel mechanistic insights compared to endpoint quantifications. When osteoclast formation was inhibited with pamidronate, the resistance remained high, comparable with the culture with only M-CSF, due to lack of resorption (Figures 5B–D). Calculation of the area under the curve (Figure 5D) showed that significant differences exist between almost all incubations with IL-1β inducing the highest resorptive activity of mature osteoclasts. No differences are present in M and PAM since no active osteoclasts were present. All the differences between the other incubations were ever seen before with the classic endpoint staining.

## 4 Discussion

In the present study, we show that ECIS can be used to accurately follow the onset and pattern of osteoclastic resorbing activity in real-time by measuring the electrical resistance of calcium phosphate–coated surfaces. We show that the decrease in resistance is due to CAP lysis by osteoclasts since addition of only M-CSF to the cultures or addition of an inhibitor of osteoclast activity, pamidronate, did not induce a decrease in resistance. On the other hand, when osteoclast activity was enhanced by adding IL-1β, a significant change in resistance, and thereby CAP resorption, was measured.

Continuous measurement of resistance by ECIS allows the analysis of the behavior of osteoclasts at any point during the culture and thereby provides a unique mechanistical look. Some significant differences were observed during this study that would not have been visible if only the end values of resistance were analyzed. An example of this is that when the cells were cultured with M-CSF and RANKL and IL-1β significantly, more CAP is lysed than when cultured with M-CSF and RANKL; the cells started resorption 1 day earlier; and the differences were no longer apparent at day 11. This means that IL-1β accelerated the initial lysis activity but not the total amount of the lysed substrate. Thus, the time point when the lysis is measured is important. In conventional methods, the cultures had to be stopped, probably at time points that are not always informative, for showing representative osteoclast activity. With the ECIS method, the cultures can continue and researchers can select time points with the relevant effect size based on real-time data.

The continuous measurement of the resorption is an important advantage and could be a great step forward in studying the direct effect of medication on bone degradation, such as bisphosphonates or cancer-inhibiting drugs with high-throughput screening methodologies, in the development of other biomimetic substrates or in co-culture studies. One might even speculate to use such coated surfaces as sensors to follow osteoclast or general mineral resorption activity *in vivo.* One could also think of using the system for quantifying the production of a matrix shown by increasing impedance values.

In summary, this novel use of the ECIS technology makes it possible to measure osteoclast activity in an open and unbiased manner during culturing, revealing hitherto unknown profiles of resorptive activity. The ECIS method is a promising high-throughput technique to investigate the resorption activity of osteoclasts from patients suffering from, for example., Paget’s disease fibrous dysplasia, osteoporosis, osteopetrosis, rheumatoid arthritis, or bone tumors with malfunctioning osteoclasts and analyze the effect of medication on osteoclast activity during culturing.

## Data Availability

The raw data supporting the conclusions of this article will be made available by the authors, without undue reservation.
